# Effects of different soil and water conservation measures on plant functional traits in the Loess Plateau

**DOI:** 10.3389/fpls.2024.1381807

**Published:** 2024-09-09

**Authors:** Gaohui Duan, Cheng Zheng, Yanmin Jiang, Chunqian Leng, Yangyang Liu, Boheng Wang, Dianjing He, Zhongming Wen

**Affiliations:** ^1^ College of Grassland Agriculture, Northwest A&F University, Xianyang, China; ^2^ Institute of Soil and Water Conservation, Chinese Academy of Sciences & Ministry of Water Resources, Yangling, China; ^3^ School of Chemical Engineering, Shandong Institute of Petroleum and Chemical Technology, Dongying, China; ^4^ First Department of Forest and Grass Comprehensive Monitoring, East China Survey and Planning Institute of National Forest and Grassland Administration, Hangzhou, China; ^5^ Ecological Engineering Department, Northwest China Survey and Planning Institute of National Forest and Grassland Administration, Xian, China

**Keywords:** ecological restoration, vegetation management, measures, plant functional traits, structural equation model (SEM)

## Abstract

Soil and water conservation measures (SWCM) have wide-ranging effects on vegetation and soil, and their effects on the ecosystem are multifaceted, with complex mechanisms. While numerous studies have focused on the impact of such measures on soil, the improvement of plant functional traits is a major factor in the ecological recovery of the Loess Plateau. This survey extensively investigated no measure plots, vegetation measure plots, and engineering measure plots in the Loess Plateau. The impact of SWCM on plant functional traits was investigated using structural equation modeling. We examined six plant functional traits—leaf dry weight (LD), specific leaf area (SLA), leaf tissue density (LTD), leaf total phosphorus (LTP), leaf total nitrogen (LTN), and leaf volume (LV)—correlated with resource acquisition and allocation. In 122 plots, we explored the effects of measures, soil, diversity, and community structure on the weighted average of plant functional traits. The findings showed substantial positive correlations between LD and SLA, LD and LV, SLA and LV, SLA and LTP, and LTP and LTN. LTD has a substantial negative correlation with LD, LTD with SLA, and LTD with LV. SWCM limits diversity, and the mechanisms by which it affects plant functional traits vary. In the structural equation model (SEM) of vegetation measures, improving community structure enhances plant functional traits, but soil factors have the greatest influence on plant functional traits in SEM engineering measures. Plant functional trait differences on the Loess Plateau result are due to differential plant responses to diverse soil properties and community structure. Vegetation measures enhance the chemical properties of plant functional traits, while engineering measures improve physical properties. The study provides a theoretical foundation for vegetation restoration and management following the implementation of diverse SWCM.

## 1 Introduction

In areas affected by soil erosion, vegetation has been extensively studied due to its significant role in erosion control and environmental improvement. Plant functional traits, which describe and evaluate the functions performed by plants in their habitat, play an important role in plant survival and reproductive strategies (Newell and Tramer, 1978; de Bello et al., 2010; Perez-Harguindeguy et al., 2016). They serve as indicators of how plants respond to changes in environmental responses (Duan et al., 2022). Numerous studies have consistently demonstrated that plant functional traits are intricately influenced by multifaceted factors, encompassing soil environment and water availability, among others (Nizamani et al., 2024; Tariq et al., 2024). For instance, afforestation and vegetation restoration projects significantly enhance soil’s physical and chemical properties, including boosting soil organic matter content, ameliorating soil structure, and augmenting its water- and nutrient-holding capacities. These improvements foster superior environmental conditions for plant growth, thereby stimulating their growth and development (Schneider et al., 2023). Furthermore, manual interventions regulate surface runoff and groundwater levels, optimizing water conditions for vegetation (Zölch et al., 2017). In arid regions, soil and water conservation forests mitigate soil water evaporation, elevate soil moisture content, and provide a stable water source for plants. This facilitates the maintenance of normal physiological processes like photosynthesis and respiration. Additionally, an ample water supply fosters leaf expansion, enhancing leaf area and photosynthetic efficiency (Ling et al., 2019). These mechanistic insights underpin a robust foundation for vegetation restoration in areas plagued by soil erosion.

Plant functional traits constitute crucial elements within the ecosystems of the Loess Plateau (de Bello et al., 2010; Perez-Harguindeguy et al., 2016; Lavorel and Grigulis, 2012; Bouallala et al., 2020). Soil and water conservation measures (SWCM) are among the most often used strategies on the Loess Plateau, with their effects on plant functional traits serving as a focus point for vegetation restoration initiatives. For example, Dharmawan et al. (2023) illuminated how both vegetation and engineering measures can bolster native plant adaptability to local climatic and edaphic conditions, thereby shaping plant functional traits. Hautier et al. (2020) reported a decline in plant species diversity under VM, attributable to alterations in the community environment and plant functional traits. Dimtsu et al. (2018) discovered that EM had no substantial effect on crop functional traits in degraded land, probably due to soil nutrients being the key limiting factor. Notably, the underlying mechanisms governing these interactions remain largely elusive (Van der Putten et al., 2013). Despite some studies focusing on individual factors, such as soil properties, water availability, runoff, and sediment transport, under various SWCM, a comprehensive analysis encompassing plant factors, abiotic components, and ecosystem dynamics at all levels remains deficient (Diaz and Cabido, 2001; Díaz et al., 2007; Faucon et al., 2017).

Soil erosion poses a pervasive global environmental challenge, often resulting in land degradation, reduced agricultural productivity, and water contamination. It also threatens both food security and ecological balance (Lal, 2001; Bewket and Teferi, 2009). The Loess Plateau in China exemplifies extreme erosion globally and serves as a significant sediment source for the Yellow River (Huo et al., 2020). To mitigate soil and water loss, a diverse array of SWCM, encompassing both engineering (EM) and vegetation measures (VM), have been extensively implemented (Shi et al., 2019; Zhao et al., 2018; Guo et al., 2007; Duan et al., 2023). These measures have led to a notable increase in vegetation cover by over 30% on the Loess Plateau and a reduction in soil and water loss by approximately 90% (Sun et al., 2014). However, the study underscores the inadequacy of focusing solely on erosion control measures, as they neglect vegetation growth dynamics and their long-term implications on runoff and sediment dynamics (Arbuckle and Roesch-McNally, 2015). Importantly, increased vegetation cover does not necessarily translate into optimized vegetation structure and functional attributes (Zhongming et al., 2010; Wen et al., 2023). The intricate pathways by which various SWCM modulate vegetation structure and function, particularly plant functional traits, and consequently influence the ecological environment of the Loess Plateau, remain largely unexplored (Jiang et al.,2019; Bekele and Drake, 2003; Duan et al., 2022).

There exists a significant knowledge gap regarding the intricate interplay between SWCM, soil properties, and plant functional traits. Currently, it remains uncertain how SWCM directly or indirectly influences these plant attributes. To bridge this gap, we employed structural equation modeling (SEM) as a tool to elucidate the causal relationships among SWCM, soil properties, community structure, plant diversity, and functional traits (Grace, 2006). This approach aids in unraveling the complexity of SWCM impacts on ecosystems (Wang et al., 2015). The present study was conducted in a soil and water conservation demonstration watershed, established 70 years ago, and a corresponding control watershed with no such measures. This study aims to explore the underlying mechanisms of the impacts of SWCM on plants and soil, especially plant functional traits. This study addressed the following research questions: (1) which plant functional traits exhibit trade-offs or synergies on the Loess Plateau; (2) what are the associations between SWCM and soil factors, community structure, diversity, and plant functional traits? (3) How can SWCM influence plant functional traits? These questions will enhance our understanding of how plant functional traits respond to changes in measures at the community level. Additionally, they will provide a theoretical foundation for future vegetation restoration efforts on the Loess Plateau.

## 2 Materials and methods

### 2.1 Site description

**Figure 1:**
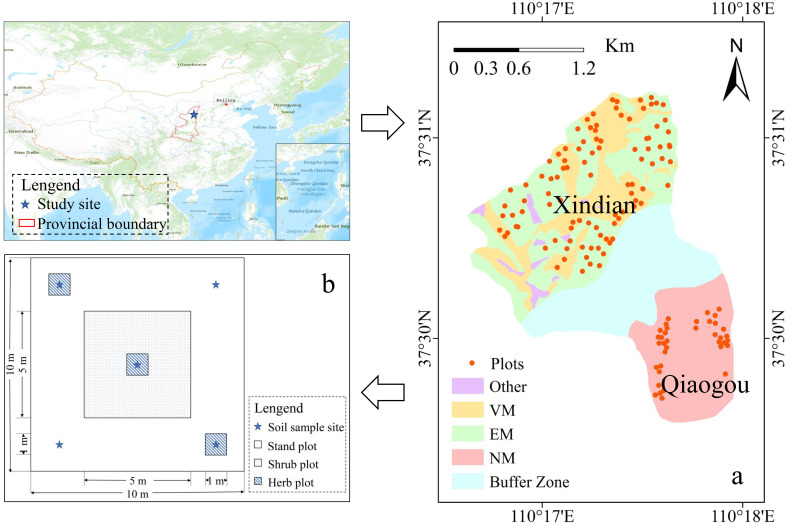
Figure **(A)** is the research area, Xindian watershed (with soil and water control measures), Qiaogou watershed (control watershed) and buffer watershed are shown, respectively; Figure **(B)** shows the position of shrub and herbaceous quadrats and the soil sampling strategy; VM, EM and NM represent vegetation measures plots, engineering measures plots and no measures plots, respectively.

The study was conducted in two adjacent watersheds, Xindiangou and Qiaogou watersheds (110° Q15 17′20″E, 37°31′09″N), located in China (**Figure 1A**). The Xindiangou watershed was established in 1952 as a SWCM national park for 72 years. In contrast, the Qiaogou watershed is characterized by its pristine and undisturbed natural environment. Both watersheds are among the most prevalent sites of soil erosion in China, situated in the northern region with a continental monsoon climate (Duan et al., 2024). The average temperature for the study period is 10.0°C, accompanied by an average annual precipitation of 430 mm from 2020 to 2024 (Wang et al., 2016), with the majority of rainfall occurring in the form of rainstorms during August. Currently, SWCM covers 80% of the Xindian watershed, resulting in a sediment reduction efficiency exceeding 97% (Duan et al., 2022).

### 2.2 Experimental design

A field survey and sampling were conducted in August 2021. We established 122 plots of 10 m × 10 m (**Figure 1B**), with a minimum distance of 10 m separating neighboring plots to prevent reciprocal influence, and one shrub plot (5 m × 5 m) and three herb plots (1 m × 1 m) were established in the plots (10 m × 10 m). The total number of plots was 122, with 36 plots in the Qiaogou watershed categorized as having no measurements (NM), 42 plots with VM (including pine, cypress, and sea buckthorn), and 44 plots with EM (such as terraces, stepwise plots, and fish scale pits) in the Xindiangou watershed.

### 2.3 Field data collection and analysis

We recorded the species names and counts within each plot, conducted individual plant surveys, and documented the vegetation coverage and average height. Unmanned aerial vehicle (UAV) photography, using a Dji Phantom 4, was employed to capture images of the plots. These images were then processed using ENVI 5.3 software to identify vegetation, followed by the use of ArcGIS 10.6 software to calculate the ratio of vegetation area to plot area, thereby determining vegetation coverage. Following the field survey, leaf samples were collected from each species. The physical characteristics of 10 fresh leaves were measured, and the remaining samples were sent to the laboratory for further analysis (Duan et al., 2023).

Soil data were collected simultaneously from each plot. Initially, soil data were gathered from five points at depths of 0–30 cm in each plot using an 8-cm auger (**Figure 1B**). Concurrently, soil bulk density, porosity, and water content were measured using a cylindrical weight sampler (100 mm^2^) (Wang et al., 2019). After impurities were removed through grinding and sieving, all soil samples were brought back to the laboratory and air-dried.

Leaf area was measured using a scanner with 10 replicates, while leaf volume was determined via the water displacement method (Pierce et al., 2013). Subsequently, the leaves were oven-dried at 65°C for 48 h to obtain the leaf dry matter mass (Guo et al., 2022). Soil bulk density was assessed using the soil core method, and soil water content was determined by the gravimetric method. Soil organic matter was quantified using the TOC analytical method, while soil nitrogen content was determined through the Kjeldahl method. Soil phosphorus was digested with H_2_SO_4_-HCIO_4_ and determined by the Bray method. For pH measurement, we prepared a 1:5 slurry by mixing soil with deionized water and utilized a Hanna HI98107 GroLine pH Tester for the measurement (Duan et al., 2023).

### 2.4 Variables

This study employed SEM to elucidate the causal relationships among various factors. SEM incorporated four independent variables (measures, soil properties, community structure, and plant diversity) and one dependent variable (plant functional traits). Three indicators, namely the tillage practice factor (C), slope (S), and SWCM factor (P), were used to characterize the measures. The soil properties were comprehensively described using nine observation variables: soil–water content (SWC), soil bulk density (BD), soil pH (pH), soil porosity (SP), soil-specific gravity (SWS), soil total nitrogen content (STN), soil organic carbon content (STC), field water capacity (FC), and soil total phosphorus content (STP). To ensure the completeness of diversity information, species richness (RIC) and species evenness (EVE) were selected. For depicting the spatial status of vegetation within the community, three community structure indicators were chosen: vegetation coverage (VC), plant height (H), and number of plants (*N*). To illuminate plant functional trait variables, the following were selected: specific leaf area (SLA), leaf volume (LV), leaf tissue density (LTD), leaf total phosphorus content (LTP), leaf dry weight (LD), leaf thickness (LT), leaf organic carbon/total nitrogen ratio (LC/N), leaf total nitrogen/total phosphorus ratio (LN/P), leaf organic carbon content (LTC), and leaf total nitrogen content (LTN) (**Supplementary Table A1**).

### 2.5 Analytical method

Random forest algorithm (RF) is a collaborative learning method that uses a voting mechanism to create final predictions (Dietterich, 2000). Initially, RF was applied to screen for crucial indices associated with plant functional traits and soil properties in the research (Kobler and Adamic, 2000; Chang and Lin, 2007; Rodriguez-Galiano et al., 2014; Pham et al., 2017; Guo et al., 2021). Subsequently, to investigate any potential relationships among plant functional traits, Pearson’s correlation analysis was employed (Huang et al., 2023). Additionally, one-way ANOVA and LSD tests were conducted to assess variations in plant functional traits under different SWCM (Wawire et al., 2021). To streamline the dataset, reduce dimensionality, and extract key information, principal component analysis (PCA) was implemented, with each variable represented by its first principal component (He et al., 2021). Lastly, SEM, a statistical model integrating causality and testing methods, was utilized to concurrently examine the effects of different measures, soil properties, vegetation structure, and diversity on plant functional traits.

All statistical analyses were performed using R version 4.1.2. The “FD” software package was utilized to calculate plant functional traits, where the results represented weighted averages. The RF algorithm was conducted utilizing the “Boruta” package in R. For structural equation modeling, we utilized the IBM AMOS 25.0. In general, a valid SEM model should fulfill the following criteria, as outlined by Wen et al. (2023): (1) a nonsignificant *χ* ^2^ test (*p* > 0.05), (2) a comparative fit index (CFI) > 0.95, (3) an incremental fix index (IFI) > 0.9, and (4) a root mean squared error of approximation (RMSEA) < 0.08.

## 3 Results

### 3.1 Filter key indicators

**Figure 2:**
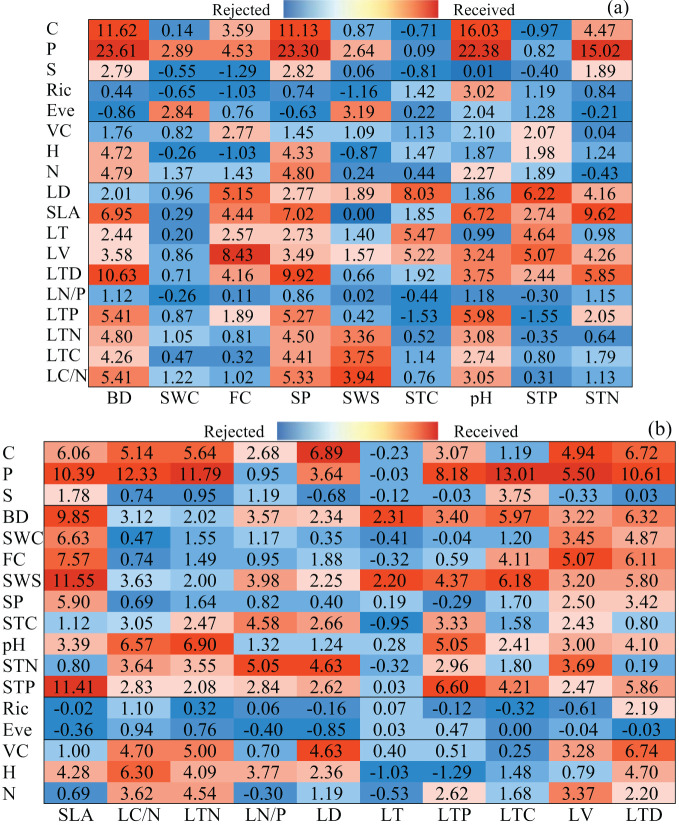
RF was employed to identify the key indicators of soil properties **(A)** and plant functional traits **(B)**. C, tillage practice factor; S, slope; P, soil and water conservation measure factor; BD, soil bulk density; SWC, soil water content; FC, field water capacity; SWS, soil-specific gravity; SP, porosity of soil; STC, soil total carbon content; pH, soil pH; STN, soil total nitrogen content; STP, soil total phosphorus content; Ric, species richness; Eve, species evenness; VC, vegetation coverage; H, plant height; *N*, total number of vegetation; SLA, specific leaf area; LC/N, ratio of carbon and nitrogen content in leaves; LTN, total nitrogen content in leaves; LN/P, ratio of nitrogen and phosphorus content in leaves; LD, leaf dry weight; LT, leaf thickness; LTP, total phosphorus content in leaves; LTC, total carbon content in leaves; LV, leaf blade volume; LTD, leaf tissue density.

In Random Forest, we incorporated additional variables to assess their influence on both plant functional traits and soil property variables, ultimately identifying the most crucial ones through a voting process. The contribution of other variables to soil property variables was deemed significant, resulting in the identification of 18 sets of statistically significant values through regression analysis (**Figure 2A**). Notably, the P group and LV group garnered the most votes, with the most groups achieving acceptability with high coefficients. Interestingly, pH was the only variable voted for in the RIC group. However, the importance values showed no correlation, prompting the rejection of all variables in the LN/P. The importance values for measures, diversity, community structure, and plant functional traits in each observed variable were 15, 3, 8, and 48, respectively. As illustrated in **Figure 2B**, we obtained 17 groups voting on plant functional traits. The STP group achieved the most votes, and the vote values for the group were indicated by the high coefficient, except for the impact on LT. The significant values of the EVE group revealed no correlation, leading to the rejection of all variables. Across observed variables, measures, diversity, community structure, and soil properties exhibited importance values of 18, 1, 16, and 53, respectively. Ranking nine dependent variables based on 74 important values, as shown in **Figure 2A**, the results were in descending order: SP (14) > BD (13) > pH (11) > FC (9) > STN (8) > STP (7) SWS (7) > STC (3) > SWC (2). Notably, SP received the most votes, primarily originating from plant functional trait variables. Conversely, SWC did not receive any votes from plant functional traits or community structure but garnered one vote each from measures and diversity. Consequently, we selected SP, BD, pH, FC, and STN as the observation variables for soil properties. In **Figure 2B**, 10 dependent variables were sorted based on 88 important values, as follows: LTD (13) LV (13) > SLA (11) > LTN (9) LD (9) LTP (9) > LC/N (8) > LN/P (7) LTC (7) > LT (2). LTD and LV garnered the greatest number of votes, with soil properties constituting the majority of votes. Notably, only LTD received a unique vote from diversity. LT received the fewest votes, with two votes from soil properties. To mitigate data collinearity, we selected LTD, LV, SLA, LTN, LD, and LTP as observation variables for plant functional traits based on voter feedback.

### 3.2 Relationships among plant functional traits

**Figure 3:**
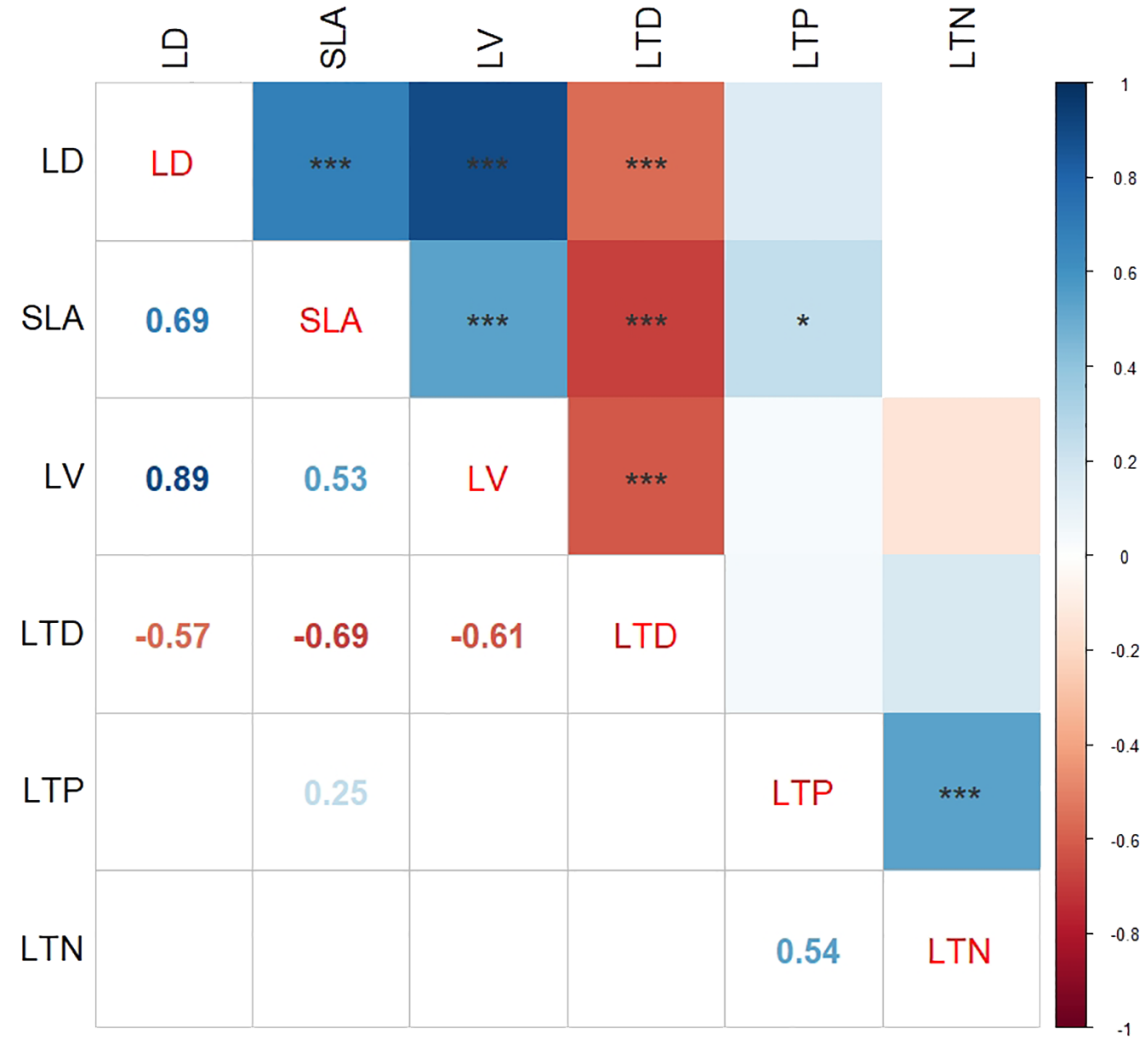
Relationships between plant functional traits. SLA, specific leaf area; LTN, total nitrogen content in leaves; LD, leaf dry weight; LTP, total phosphorus content in leaves; LV, leaf blade volume; LTD, leaf tissue density. * indicates p < 0.05; *** indicates p < 0.001.

The correlation heat map presented in **Figure 3** illustrates the relationships among plant functional trait variables. Specifically, LD exhibited a strong positive correlation with both SLA (*r* = 0.69, *p* < 0.001) and LV (*r* = 0.89, *p* < 0.001), suggesting a close association between these traits. Furthermore, the positive correlation between SLA and LV (*r* = 0.53, *p* < 0.001) and SLA and LTP (*r* = 0.25, *p* < 0.05) was consistent, indicating a coherence in the functional traits. Notably, a significant positive correlation was also observed between LTP and LTN (*r* = 0.54, *p* < 0.001). Conversely, LTD displayed a negative correlation with LD (*r* = − 0.57, *p* < 0.001), SLA (*r* = − 0.69, *p* < 0.001), and LV (*r* = − 0.61, *p* < 0.001), suggesting an inverse relationship between these variables.

### 3.3 Plant functional traits under different measures

**Figure 4:**
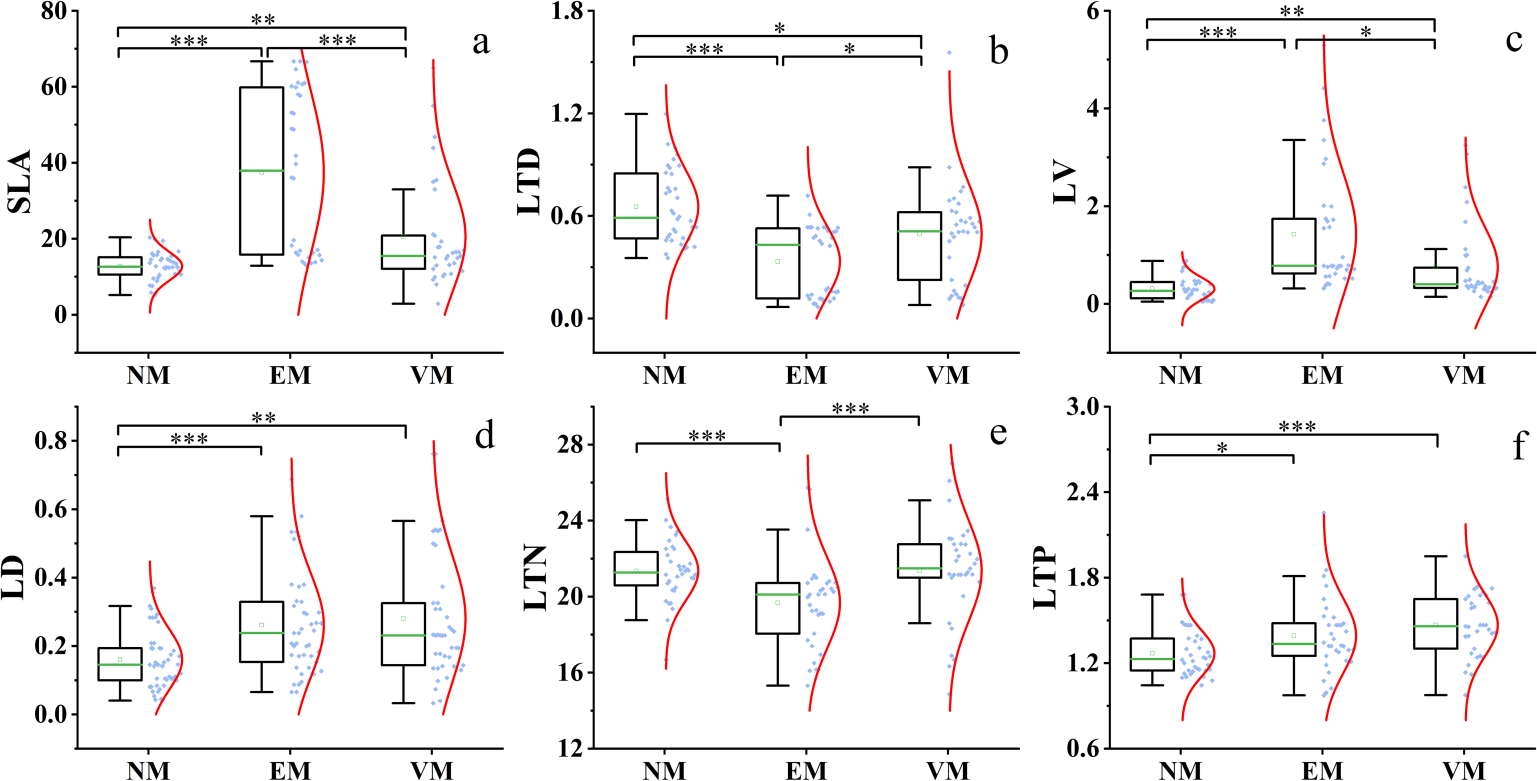
Plant functional traits under different SWCM. NM, no measure plot; VM, vegetation measure plot; EM, engineering measure plot; SLA **(A)**, specific leaf area; LTD **(B)**, leaf tissue density; LV **(C)**, leaf blade volume; LD **(D)**, leaf dry weight; LTN **(E)**, total nitrogen content in leaves; LTP **(F)**, total phosphorus content in leaves.

The SWCM significantly improved plant functional traits (**Figure 4**). Most plant functional traits varied significantly in response to the implemented measures. Generally, in EM plots, SLA, LV, and LD indices increased significantly, while the LTN index decreased slightly as compared to NM plots. SLA, LV, LD, LTN, and LTP indices showed a slight increase in VM plots. Additionally, the probability distribution of SLA, LTD, and LD indices was more dispersed than those of LV, LTN, and LTP indices, and the standard deviations followed a similar trend. Most plant functional traits under various SWCM differed significantly from NM plots. Specifically, SLA, LTD, and LV exhibited significant differences across various measures. There was no significant difference between EM and VM in LD and LTP (**Figures 4D, F**) or NM and VM in LTN (**Figure 4E**).

### 3.4 Factors affecting plant functional traits (no measures, vegetation measures, engineering measures)

In the following table, PCA revealed that the first principal component accounted for 61.72%, 48.18%, 51.43%, 46.24%, and 40.69% in measures, diversity, community structure, soil properties, and plant functional traits, respectively (**Table 1**).

**Table 1:** PCA results for the independent variables and dependent variables (with all plots).

Measures (61.72%)		Community structure (51.43%)		Soil (46.24%)		Functional traits (40.69%)	
C	0.638	VC	0.638	BD	0.412	LTD	0.367
P	0.645	H	0.471	FC	− 0.128	LTN	− 0.485
S	0.327	N	0.412	SP	0.411	SLA	− 0.367
				pH	− 0.257	LV	0.137
Plant diversity (48.18%)				STN	0.108	LD	0.527
Richness	0.672					LTP	0.209
Evenness	0.673						

**Figure 5:**
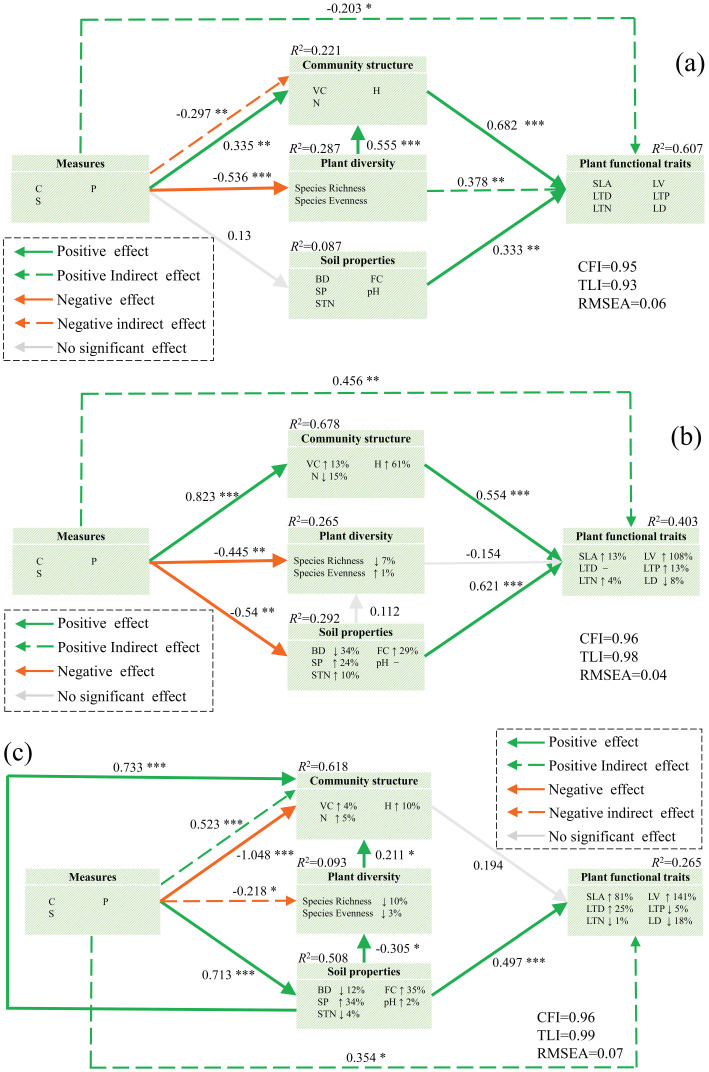
Effects of various variables on plant functional traits. **(A)** NM plots, **(B)** VM plots, and **(C)** EM plots. Green and orange indicate positive and negative effects, respectively. The solid and dashed lines indicate direct and indirect effects, respectively, and the gray arrows represent insignificant effects (*p* > 0.05). The up and down arrows after the indicators indicate lifting or lowering in different measures. Path standardized coefficients (β) are marked on the arrows, and significance levels are categorized as follows: ^*^ *p* < 0.05; ^**^ *p* < 0.01; ^***^ *p* < 0.001. C, tillage practice factor; S, slope; P, soil and water conservation measure factor; BD, soil bulk density; FC, field water capacity; SP, porosity of soil; pH, soil pH; STN, soil total nitrogen content; Ric, species richness; Eve, species evenness; VC, vegetation coverage; H, plant height; N, total number of vegetation; SLA, specific leaf area; LTN, total nitrogen content in leaves; LD, leaf dry weight; LTP, total phosphorus content in leaves; LV, leaf blade volume; LTD, leaf tissue density.

SEM was developed to investigate the impact of measures, diversity, community structure, and soil properties on plant functional traits. The measurement data supported the hypothesized model. The results revealed that the contribution rates were 22.1% for community structure, 28.7% for diversity change, 8.7% for soil properties, and 60.7% for plant functional traits change in the NM model. The model indicated that measures had significant direct effects on community structure (*p* < 0.01) and diversity (*p* < 0.001), and plant diversity significantly influenced community structure (*p* < 0.001). Community structure (*p* < 0.001) and soil properties (*p* < 0.01) directly influenced plant functional traits. Furthermore, measures had indirect effects on community structure (*p* < 0.01), and both measures (*p* < 0.05) and diversity (*p* < 0.01) indirectly influenced plant functional traits through community structure (**Figure 5A**). The results revealed contribution rates of 67.8%, 26.5%, 29.2%, and 40.3% for community structure, diversity, soil properties, and plant functional traits, respectively. VM significantly influenced community structure (*p* < 0.001), diversity (*p* < 0.01), and soil properties (*p* < 0.01). Community structure (*p* < 0.001) and soil properties (*p* < 0.001) directly influenced plant functional traits. VM had a significant indirect effect on plant function (*p* < 0.01) via community structure (**Figure 5B**). **Figure 5C** illustrates that structural equations account for 61.8%, 0.093%, 50.8%, and 26.5% of community structure, diversity, soil properties, and plant functional traits, respectively. According to regression weight estimation, EM significantly influenced both community structure (*p* < 0.001) and soil properties (*p* < 0.001). Soil property factors significantly impacted community structure (*p* < 0.001), diversity (*p* < 0.05), and plant functional traits (*p* < 0.001). Plant diversity strongly impacts community structure (*p* < 0.05). Additionally, EM had significant indirect effects on diversity (*p* < 0.05), community structure (*p* < 0.001), and plant functional traits (*p* < 0.05) via soil properties.

## 4 Discussion

### 4.1 Trade-offs and synergies between plant functional traits

Investigating the correlation among plant functional traits is pivotal for understanding how plants adapt to environmental fluctuations within ecological processes (Nicotra et al., 2010; Chillo et al., 2018; Wang and Ali, 2020). Over time, plants interact with their environment, evolving adaptive traits synergies to thrive and reproduce under given conditions (Oscarson, 2000). The leaf economic spectrum theory, which integrates these traits, emphasizes how plant functional traits vary under environmental stress, spanning from resource acquisition to protection (Cornwell et al., 2010). The coordination of plant functional traits arises from biophysical trade-offs inherent in ecological strategies (Lavorel et al., 2011). For instance, SLA exhibits a positive correlation with LTP and LD, signifying enhanced photosynthetic capacity and nutrient turnover (Shu-Xia et al., 2016). This implies that higher SLA leads to increased photosynthetic efficiency, enabling plants to synthesize organic matter more efficiently, resulting in faster growth rates, consistent with the global leaf economic spectrum (Poorter and Evans, 1998). Furthermore, LTN and LTP display a positive linear relationship, indicating their concerted changes in vegetation on the Loess Plateau. This provides a robust foundation for sustained plant growth and development. LTN and LTP reflect the plant’s capacity to utilize these vital nutrients, which increase with SWCM (Huang et al., 2023). The significant negative correlation between LTD and LD and LV is primarily attributed to SWCM mechanisms, which enhance soil moisture retention and subsequently elevate leaf water content (Zhang et al., 2009). Conversely, the arid conditions prevalent on the Loess Plateau exert stress on vegetation, necessitating a reduction in leaf volume and area to minimize water consumption (Wang et al., 2011). This observation aligns with the notable negative relationship between LTD and SLA, indicating that under harsh environmental conditions, plants allocate greater amounts of synthesized dry matter to leaf structures (Yan et al., 2016). This strategy enhances drought resistance by facilitating the diffusion of internal moisture to the leaves, as demonstrated by Duan et al. (2023).

### 4.2 Effect of measures on plant functional traits in no measure plots

The model underscores the indirect influence of measures on plant functional traits via community structure and diversity in NM plots. Furthermore, community structure and soil properties exert direct effects on plant functional traits, whereas plant diversity mediates an indirect effect. Zheng et al. (2017) conducted plot-based experiments, demonstrating that changes in soil nutrients resulting from N and P addition accounted for over 50% of the variation in plant functional traits. Similarly, Chaturvedi et al. (2021) identified soil moisture as the primary regulator of plant functional trait variation (Li et al., 2021). Furthermore, Huang et al. (2023) emphasized the significance of community structure, with a primary influence on plant functional traits such as SLA, LD, LTP, and LTN, through selective thinning experiments. Notably, measures imposed strongly negatively impacted diversity, highlighting the primary effect of artificial interventions in Loess Plateau ecosystems as the reduction of diversity (Huang et al., 2023). Concurrently, measures significantly and directly influenced community structure, exemplified by SWCM’s capacity to mitigate soil erosion, thereby preserving plant communities (DeKeyser et al., 2003). Introducing locally adapted species can also enhance habitats and enrich plant communities (Terborgh, 1973). In summary, SWCM has a multifaceted impact on plant communities, intricately linked to soil–vegetation interactions.

### 4.3 Effect of vegetation measures on plant functional traits

VM are extensively utilized for soil and water conservation, significantly influencing various vegetation variables. The results of SEM demonstrated that VM exerts considerable indirect effects on plant functional traits through alterations in community structure. Additionally, soil properties directly impact plant functional traits. When compared to the NM plots, VM improved community structure and diversity by 1.12 and 0.091, respectively, while decreasing soil properties by 0.67. Notably, the influence of community structure on plant functional traits diminished by 0.128, whereas the impact of soil properties on plant functional traits increased by 0.288. Furthermore, the indirect effects of VM on plant functional traits increased by 0.659. In the study area, measures such as afforestation have bolstered forest coverage and fortified grassland protection, particularly in the remediation of degraded land ecosystems (Yurui et al., 2021). These interventions mitigate herbaceous plant damage during heavy rainfall and provide shade to plants during dry seasons, facilitating water retention (Jian et al., 2015; Gardiner et al., 2016; Wu et al., 2023). Consequently, the improved microclimate fosters vegetation growth, leading to enhanced community structure and diversity (Yang et al., 2011). However, researchers have observed that excessive vegetation restoration through VM can have adverse effects, with soil drying emerging as a prominent consequence due to excessive soil moisture consumption (Roa-Garcia et al., 2011; Zhang et al., 2018). Ultimately, these factors collaborate to shape the functional traits of plants.

VC and H increased by 13% and 61%, respectively, whereas N decreased by 15%. The application of VM led to varying degrees of enhancement in plant height, coverage, and significant improvements in community structure. This phenomenon may be ascribed to the measures’ ability to bolster ecosystem functioning, fostering more stable and conducive environments for plant growth, thereby influencing plant community composition (Duan et al., 2023). Conversely, the plant diversity index, RIC, decreased by 7%, while EVE increased by 1%. Long-term experiments on nitrogen addition in grasslands have revealed that heightened soil nitrogen content can lead to reduced species diversity, potentially due to vegetation’s inability to adapt to acidified soils (Fang et al., 2012). Among the factors contributing to grassland diversity decline are differing levels of adaptability to environmental amelioration, variations in nutrient acquisition and utilization strategies among species, and habitat fragmentation stemming from land use changes (Niedrist et al., 2009). Among soil properties, FC, SP, and STN increased by 29%, 24%, and 10%, respectively, while BD decreased by 34% and pH remained unchanged. This and other similar studies have shown that VM enhances soil physical properties (Gu et al., 2019; Davari et al., 2020; Kooch et al., 2020). It has been observed that the extensive planting of trees and shrubs can significantly improve the physical quality of soil, subsequently fostering an increase in photosynthetic capacity and stomatal conductance in plant leaves (Black et al., 1991; Bond, 2000). Additionally, plant functional traits such as SLA, LV, LTP, and LTN increased, while LTD remained stable. This evidence supports the notion that improved soil quality promotes rapid plant growth and efficient resource acquisition (Volkmann et al., 2016). In conclusion, SEM analysis revealed a notable enhancement in community structure and soil properties with VM plots, ultimately influencing plant functional traits.

### 4.4 Effect of engineering measures on plant functional traits

The SEM analysis of EM demonstrated a statistically significant positive correlation between the measures and soil properties, indirectly influencing plant functional traits. This linkage arises from the core objective of EM, which is aimed at mitigating soil erosion through modifications to the terrain. As a result, EM facilitates the retention of soil moisture and nutrients, leading to substantial and sustained enhancements in both soil properties and plant functional traits (Hudson, 2015). In comparison with the control plot, the effect of EM on soil properties increased by 0.7, and the negative effect on community structure increased by 1.383. Additionally, the effects of soil properties on community structure and plant functional traits increased by 0.733 and 0.164, respectively. Furthermore, the indirect effects on community structure and plant functional traits increased by 0.82 and 0.557, respectively. The observed findings can be attributed to several factors: (1) The use of EM altered the terrain structure, resulting in changes in water and air temperature, which subsequently modified soil characteristics and structure. These alterations proved conducive to vegetation growth (Duan et al., 2023). (2) The varying water and air temperature conditions induced by EM impacted soil properties and community structure. Furthermore, enhancements in soil quality influenced plant diversity and competitive interactions among species, leading to intricate consequences on plant functional traits (Dubuis et al., 2013; Pellegrini et al., 2018). (3) These influences simultaneously altered plant functional traits by modifying both soil conditions and community structure.

### 4.5 Prospects for the influence of soil and water conservation measures on plant functional traits

The influence of SWCM on plant functional traits is intricate and interdependent. Firstly, amidst the intensifying impacts of global climate change, the significance of SWCM in mitigating extreme weather events, such as floods and droughts, becomes paramount. These measures can potentially aid plants in adapting to more variable climatic conditions by enhancing soil water retention and optimizing plant water use efficiency. Consequently, we foresee an augmented influence of SWCM on plant functional traits in the foreseeable future. Secondly, advancements in ecological and plant biological research will facilitate a deeper comprehension of plant functional traits, enabling more accurate predictions of how SWCM affects plant functioning. For instance, research may reveal that specific SWCM exerts a stronger influence on certain plant functional traits (e.g., photosynthetic efficiency and fertilizer use efficiency). Moreover, future SWCM may prioritize ecosystem integrity and synergy, encompassing more intricate plant communities and comprehensive land management strategies. In this context, SWCMs are anticipated to exert more complex and diverse effects on plant functional traits. To gain a deeper understanding and accurate forecasting of the impacts of soil and water conservation techniques on plant functional traits, continuous research, and monitoring are imperative. Furthermore, to effectively safeguard our land resources and biological environment, it is crucial to adapt to future changes and continually refine and optimize these techniques.

## 5 Conclusions

The study reveals synergistic interactions among plant functional traits within different SWCM and identifies trade-offs between specific traits. It also highlights that SWCM has varying impacts on plant functional traits, with some enhancing certain traits while reducing plant diversity. The results suggest that VM and EM affect plant function traits through changes in community structure and soil properties, respectively. While both measures (VM and EM) can improve plant traits, VM is more effective in long-term ecological restoration due to its ability to reduce soil erosion and create better habitats for plants. Therefore, VM should be prioritized in areas with lower erosion risk for future interventions.

## Data Availability

The data that support the findings of this study are available from the corresponding author upon reasonable request.
