# Supplementary material for: Effects of different soil and water conservation measures on plant functional traits in the Loess Plateau
Source: Front Plant Sci. 2024 Sep 9;15:1381807. doi: 10.3389/fpls.2024.1381807 (PMC11418278; doi:10.3389/fpls.2024.1381807)
Supplement: Supplementary file 1 [file Table1.docx]

Table. A.1 Abbreviation, units, plant trait measured.

| Traits | Abbreviation | Unit | Formula |
| --- | --- | --- | --- |
| Vegetation coverage | VC | no units | Total plant cover in the plot |
| Plant height | H | m | Average height of plants in the plot |
| Total number of vegetation | N | no units | Total number of plants in the plot |
| Species richness | RIC | no units | The number of species in each plot |
| Species evenness | EVE | no units | Pielou evenness |
| Specific leaf area | SLA | cm^2^g^-1^ | Specific leaf area |
| Leaf blade volume | LV | cm^3^ | Mean leaf volume in the plot |
| Leaf tissue density | LTD | gcm^-3^ | Ratio of leaf dry weight to leaf volume |
| Total phosphorus content in leaves | LTP | mg g^-1^ | Total phosphorus content of leaves in each plot |
| Total nitrogen content in leaves | LTN | mg g^-1^ | Total nitrogen content of leaves in each plot |
| leaf dry weight | LD | g | The weight of the leaves after drying |
| Soil bulk density | BD | gcm^-3^ | The weight of soil in a unit volume |
| Field water capacity | FC | no units | The content of water in soil |
| Porosity of soil | SP | no units | Soil pore volume/The soil volume |
| Soil pH | pH | no units | Soil pH in the plot |
| Soil total nitrogen content | STN | mg g^-1^ | The content of total nitrogen of soil |
| Tillage practice factor | C | no units | Equal to the ratio of soil loss in specific vegetation and management plots to soil loss in standard plots when other conditions are the same |
| Slope | S | no units | Mean angle of the site to the horizontal |
| Soil and water conservation measures factor | P | m | Equal to the ratio of soil loss in the current plot to soil loss in the standard plot when other conditions are the same |
